# Thermal Shock Performance of DBA/AMB Substrates Plated by Ni and Ni–P Layers for High-Temperature Applications of Power Device Modules

**DOI:** 10.3390/ma11122394

**Published:** 2018-11-28

**Authors:** Chanyang Choe, Chuantong Chen, Seungjun Noh, Katsuaki Suganuma

**Affiliations:** 1 Department of Adaptive Machine Systems, Graduate School of Engineering, Osaka University, Osaka 5670047, Japan; cychoe@eco.sanken.osaka-u.ac.jp (C.C.); sjnoh@eco.sanken.osaka-u.ac.jp (S.N.); 2 The Institute of Scientific and Industrial Research, Osaka University, Osaka 5670047, Japan; suganuma@sanken.osaka-u.ac.jp

**Keywords:** DBA, AMB, Ni electroplating, Ni–P electroless plating, cracking, grain boundary sliding, thermal shock test, roughness, metallization, reliability

## Abstract

The thermal cycling life of direct bonded aluminum (DBA) and active metal brazing (AMB) substrates with two types of plating—Ni electroplating and Ni–P electroless plating—was evaluated by thermal shock tests between −50 and 250 °C. AMB substrates with Al_2_O_3_ and AlN fractured only after 10 cycles, but with Si_3_N_4_ ceramic, they retained good thermal stability even beyond 1000 cycles, regardless of the metallization type. The Ni layer on the surviving AMB substrates with Si_3_N_4_ was not damaged, while a crack occurred in the Ni–P layer. For DBA substrates, fracture did not occur up to 1000 cycles for all kind of ceramics. On the other hand, the Ni–P layer was roughened and cracked according to the severe deformation of the aluminum layer, while the Ni layer was not damaged after thermal shock tests. In addition, the deformation mechanism of an Al plate on a ceramic substrate was investigated both by microstructural observation and finite element method (FEM) simulation, which confirmed that grain boundary sliding was a key factor in the severe deformation of the Al layer that resulted in the cracking of the Ni–P layer. The fracture suppression in the Ni layer on DBA/AMB substrates can be attributed to its ductility and higher strength compared with those of Ni–P plating.

## 1. Introduction

Power electronic modules, such as converter and inverter systems, have been widely used in transportation, including electric vehicles, aircraft, and high-speed railroads. Usually, the power module simply consists of five main components: wires, semiconductor devices, joints, substrates, and heat sink. With the increasing use of wide-bandgap (WBG) semiconductor devices, such as silicon carbide (SiC) and gallium nitride (GaN), which provide great opportunities to develop power electronic systems with increased power densities, high reliability in extreme environments, and higher integration, the development of high-temperature-operating power devices allows for the use of power electronic modules at high temperatures (>250 °C) [1,2]. In this case, large thermal stress occurs in the power electronic module due to coefficients of thermal expansion (CTE) mismatch in multiple layers, leading to a reliability issue for its high-temperature application.

To dissipate the heat that induces power electronic module failure, a power electronic substrate plays an important role. A power electronic substrate is located between a semiconductor die and heat sink and transfers the heat generated in the semiconductor to cooling plates [3,4,5]. Direct bonded aluminum (DBA) and active metal brazing (AMB) substrates have been considered as the most promising substrates for power electronic modules due to their good thermal conductivity, low electrical resistance, and high insulation voltage [6,7]. The advantage comes from the metal/ceramic/metal sandwich structure of DBA/AMB substrates. DBA/AMB basically has a ceramic insulator plate composed of materials such as Al_2_O_3_, AlN, and Si_3_N_4_. Both sides of an insulator plate are metalized by aluminum (Al) or copper (Cu) to function as a thermal and electrical conductor layer [8]. Such a metal layer enables not only improved thermal conductivity but also creates electrical circuits on an insulator plate.

However, the sandwich structure of DBA/AMB substrates also results in a large amount of thermomechanical stress induced by the difference in the CTE between metal and ceramic in the sandwich structure under high temperatures [5,9,10,11]. The repeated thermomechanical stress finally induces the fracture of DBA/AMB substrates [12]. Thermomechanical stress in DBA/AMB substrates at high temperatures depends on the material properties of the ceramic and metal components. In addition, the Ni metallization, fabricated by two principally different process techniques—the electroplated Ni plating and electroless Ni–P plating—is most generally plated on the surface of the DBA/AMB substrates as a metallization layer to protect the oxidation of Cu and Al. Additionally, electroplated Ni plating and electroless Ni–P plating also have different mechanical properties. Ni electroplating has good ductility and high tensile strength [13,14], while electroless Ni–P coating has good hardness and a simple manufacturing process because of its chemical reduction process without an electrical connection [15,16,17,18,19,20,21,22].

In the investigated case, DBA/AMB substrates were composed of various material designs, including three types of ceramic plates and two types of metal, as mentioned earlier. Additionally, Ni metallization was fabricated by two principally different process techniques: electroplated Ni plating and electroless Ni–P plating. The thermal shock performance of the DBA/AMB substrates as well as the Ni metallization layer determines the whole lifespan of the power electronic module [9,21,22]. Therefore, the material design of the DBA/AMB substrates became an important issue, since it determines their thermal cycling reliability in the power electronic module. However, the thermal cycling reliability of Ni-plated DBA/AMB substrates with various ceramics has rarely been studied.

In this study, a thermal shock cycling test of DBA/AMB substrates with various ceramics plated with Ni and Ni–P layers was implemented to investigate their high-temperature reliability for a WBG power electronic module. The cracking and roughening of the Ni–P layer on the DBA/AMB substrates were evaluated by field-emission scanning electron microscopy (FE-SEM), scanning acoustic tomography (SAT), and laser profile microscopy. The fracture suppression mechanism in the Ni layer on the DBA/AMB substrate was also examined with a stress analysis by finite element method (FEM) simulation.

## 2. Materials and Methods

**Figure 1:**
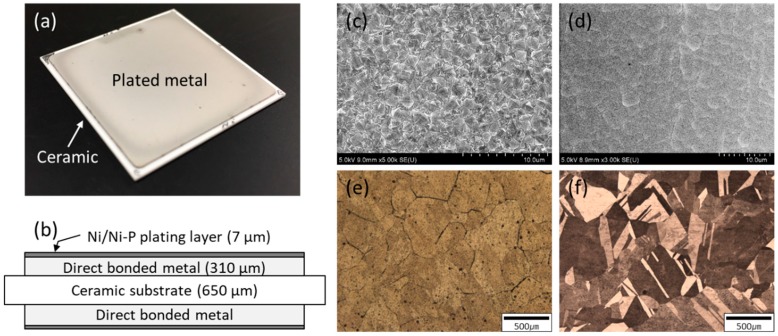
(**a**) Macrograph of a typical substrate specimen, (**b**) cross-section diagrams of the DBA/AMB substrates plated by Ni and Ni–P layers, surface morphology of Ni (**c**) and Ni–P (**d**) layer on the substrates, and surface microstructure of Al (**e**) and Cu (**f**) of the DBA/AMB substrates.

In this work, DBA and AMB substrates were fabricated by Mitsubishi Materials Co., Japan. Table 1 shows all the material combinations of DBA/AMB substrates, such as substrate types and plated layers. The dimensions of the ceramics of Al_2_O_3_, AlN, and Si_3_N_4_ were 32 × 32 × 0.65 mm and the dimensions of the Al and Cu metal were 30 × 30 × 0.31 mm, which was bonded on both sides of the ceramic plate. The Ni metallization layer on the DBA/AMB substrates was conducted by electro and electroless plating methods. The electroplated Ni film was deposited at the current density of 2 A/cm^2^. Ni–P electroless deposition was plated at 85 °C for pH 6.4. It was confirmed that the Ni–P plating layer contained 1 wt% phosphorus. Two different metallization times were respectively adjusted to achieve an average thickness of around 7 µm. Figure 1a shows the photo image of the DBA substrate coated with a Ni–P layer with the electroless plating. The dimensions and structure of the DBA/AMB substrate coated with a Ni metallization layer are shown in Figure 1b. Figure 1c,d show the SEM image of the surface of the Ni metallization layer by electro and electroless plating, respectively. The electroplated Ni layer had a pyramid-like crystal morphology, which is generally observed [23]. On the other hand, the Ni–P electroless plating layer had a smoother surface than the Ni electroplating layer. Figure 1e,f show the microstructure of Al and Cu with a few hundred micrometer-sized grains in the DBA and AMB substrates, respectively. Two samples per material combination were fabricated to check the experiment’s reproducibility.

To evaluate the high-temperature reliability of the DBA/AMB substrates with Ni electroplating and Ni–P electroless plating layers, a thermal shock cycling test between −50 and 250 °C was performed for 1000 cycles by using a thermal shock chamber (TSE-110-A-S, ESPEC Corp., Osaka, Japan). The dwelling time at the upper and lower temperatures was 30 min. Regarding the industry standards for the harsh environmental testing of microelectronics [24,25], the temperature range of a thermal shock test is set between low temperatures of −50 to −40 °C and high temperatures of 125 to 150 °C. However, advanced SiC power electronic modules are generally expected to be used at a temperature of 250 °C. For that reason, the upper temperature of 250 °C was chosen for this study. In addition, two samples per type were tested after 100 cycles and it was confirmed that the two samples had reproducibility and could achieve the same results.

To characterize the fracture in the metallization and substrates during the thermal shock cycling test, the thermally aged specimens were taken out at 0, 50, 100, 200, 600, and 1000 cycles. The surface variation of the electro and electroless plating layers was analyzed by FE-SEM (SU8020, HITACHI, Tokyo, Japan) and SAT (FineSAT FS300, HITACHI, Japan). The surface roughness of the plated specimens was measured using laser profile microscopy (VK-9510, Keyence Corp., Osaka, Japan). In order to investigate the failure mechanism, the cross-section of the aged plating layer was prepared by focused ion beam (FIB, FIB-2100, HITACHI, Japan) milling and observed using FE-SEM.

To understand the thermal stress distribution, the stress distribution in Ni- and Ni–P-plated DBA/AMB substrates at a high temperature of 250 °C during a thermal shock cycling test was investigated by a two-dimensional FEM simulation. The model was fabricated by commercial FEM code (ANSYS 15.0). The Al and Cu metals in DBA/AMB substrates were considered as a polycrystalline face centered cubic structure consisting of hypothetical grains of (111), (110), and (001) orientation.

**Figure 2:**
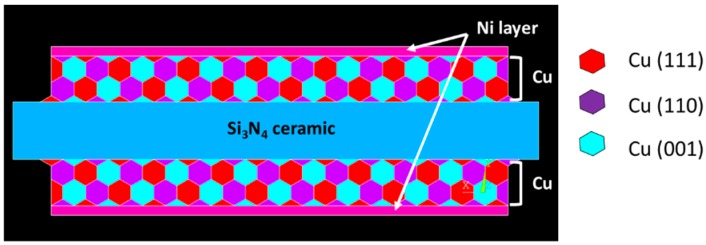
FEM model for thermal stress distribution simulation of an AMB substrate with Si_3_N_4_.

Figure 2 representatively shows the FEM model of a Ni-plated AMB substrate with Si_3_N_4_ ceramic, where the Cu metal consisted of Cu (111), Cu (110), and Cu (001) orientations. In this simulation, the angular point of the model was fixed in the X, Y directions. The stress-free temperature for the model was assumed to be 25 °C. FEM was used for elastic–plastic deformation analysis. Because there was no additional stress in this model, it was considered that the FEM analysis exhibited a plane stress state. The properties of the materials used for this model of plated DBA/AMB substrates are summarized in Table 2.

## 3. Results and Discussion

### 3.1. Thermal Shock Behavior

#### 3.1.1. Active Metal Brazing (AMB)

**Figure 3:**
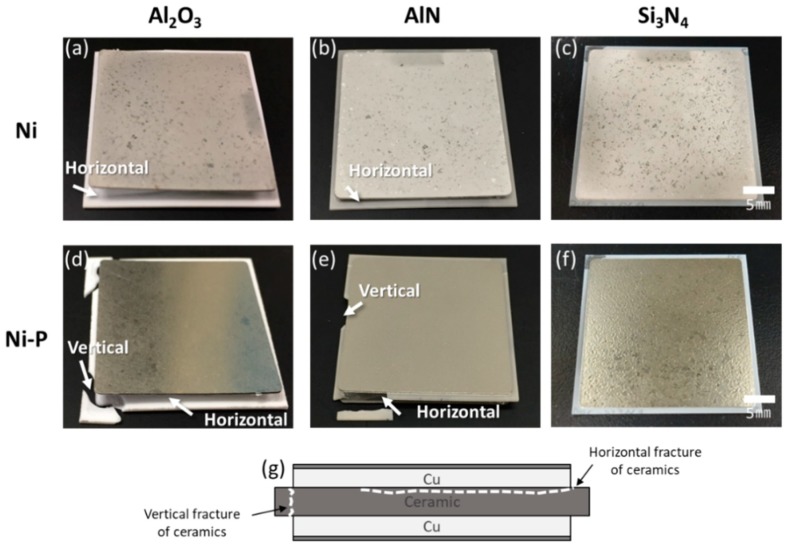
Destructed AMB substrates with Al_2_O_3_ (**a**,**d**) and AlN (**b**,**e**) after 10 cycles, nondestructed AMB substrates with Si_3_N_4_ (**c**,**f**) after 1000 cycles plated with Ni (**a**–**c**) and Ni–P (**d**–**f**), and (**g**) fracture pattern of AMB substrates.

Figure 3 shows Ni- and Ni–P-plated AMB substrates with Al_2_O_3_ and AlN ceramic after 10 cycles and those with Si_3_N_4_ ceramic after 1000 cycles. AMB substrates with Al_2_O_3_ and AlN ceramic fractured regardless of the type of metallization. The fracture of Al_2_O_3_ and AlN ceramic in the AMB substrates occurred along vertical and horizontal directions, as shown in Figure 3g. The horizontal fracture occurred inside the Al_2_O_3_ and AlN ceramic, while the interface between Cu and the ceramic plates was still bonded together. On the other hand, only AMB substrates with Si_3_N_4_ survived until 1000 cycles, regardless of the metallization type. Although Si_3_N_4_ ceramic had the biggest CTE mismatch with Cu among the three types of ceramic, as shown in Table 2, the longer thermal cycling lifetime of the Si_3_N_4_ substrate was confirmed, which agrees with a report in a recent study [26]. This might be due to Si_3_N_4_ having a larger fracture toughness of 7 MPa·m^1/2^, which describes the ability of a material to resist crack extension and fracture, than those of the other ceramic substrates. Among the three types of AMB substrates, AMB substrates with Si_3_N_4_ showed the best thermal shock resistance.

**Figure 4:**
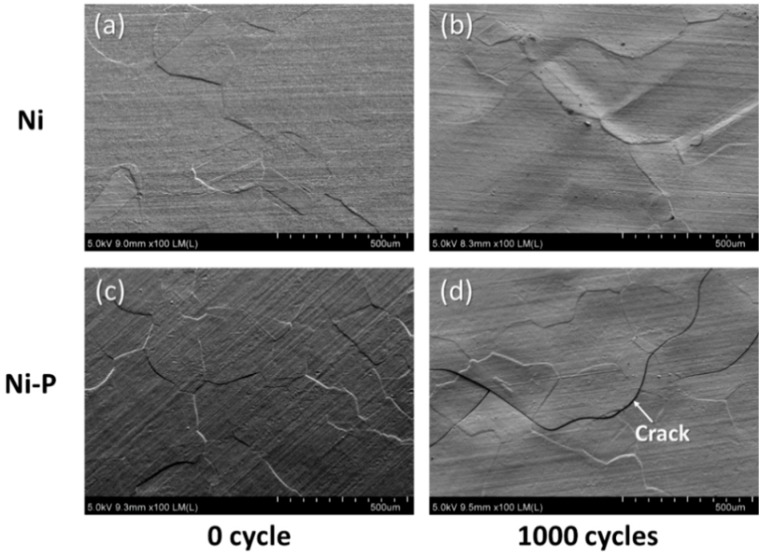
Surface variation of Ni (**a**,**b**) and Ni–P (**c**,**d**) layers on AMB substrates with Si_3_N_4_ before and after 1000 cycles.

**Figure 5:**
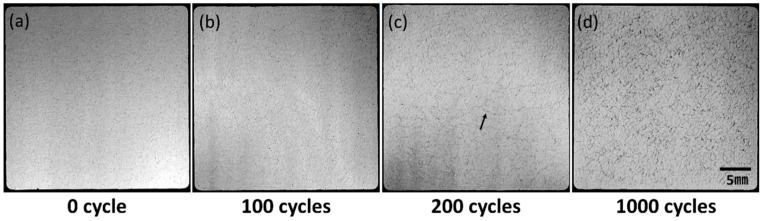
Scanning acoustic tomography (SAT) observation of the surface morphology of the Ni–P layer on AMB substrates with Si_3_N_4_ after different thermal shock tests: 0, 50, 100, and 1000 cycles.

Figure 4 shows SEM images of the surface of the Ni electroplating and Ni–P electroless plating layers on AMB substrates with Si_3_N_4_ at the initial state and after 1000 cycles. A thin crack was observed in the Ni–P electroless plating layer, as shown in Figure 4d, which was not visible through visual inspection, see Figure 3f, while a crack did not occur in the Ni electroplating layer after 1000 cycles. Crack generation and propagation of the Ni–P layer on the AMB substrates with Si_3_N_4_ were investigated by SAT observation. Figure 5 shows SAT surface images of the Ni–P layer on AMB substrates with Si_3_N_4_ during a thermal shock cycle test of 1000 cycles. The dark area in the SAT surface images indicates surface defects such as cracks, voids, and roughening, which were made by the diffused reflection of the acoustic wave at the surface defect. The surface defects were observed in the SAT images, although they cannot be definitively distinguished between cracking and roughening. The surface defects gradually increased with thermal shock cycles.

#### 3.1.2. Direct Bonded Aluminum (DBA)

**Figure 6:**
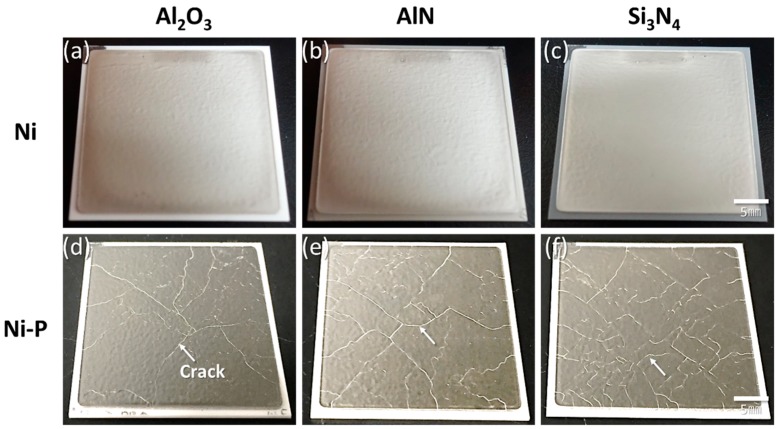
DBA substrates with Al_2_O_3_, AlN, and Si_3_N_4_ after 1000 cycles, plated with Ni (**a**–**c**) and Ni–P (**d**–**f**).

All DBA substrates survived until 1000 cycles, regardless of the ceramic material used, as shown in Figure 6. The three types of ceramic in the DBA were the same as those in the AMB, and the Ni and Ni–P plated on the DBA substrates also involved the same process as that plated on AMB substrates. However, unlike the DBA substrates, the AMB substrates with Al_2_O_3_ and AlN were fractured. This indicated that the thermal stress which occurred in the DBA with Al_2_O_3_ and AlN ceramics should be smaller than that of the AMB. The thermal stress dependence of metal types in DBA/AMB substrates was studied by Hamilton et al. [4]. It was reported that DBA substrates have lower stress than AMB substrates due to the lower yield strength of aluminum. This might be attributed to DBA having longer thermal shock resistivity compared to AMB in spite of the bigger CTE mismatch of aluminum in DBA.

**Figure 7:**
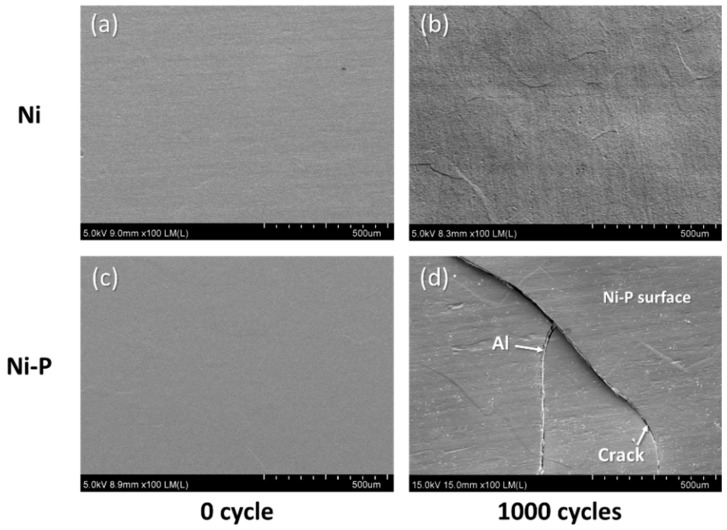
Surface morphology of Ni (**a**,**b**) and Ni–P (**c**,**d**) layers on DBA substrates with Si_3_N_4_ before and after 1000 cycles.

On the other hand, the Ni–P layer on the DBA substrates was cracked after 1000 cycles, regardless of the type of ceramic plate, as shown in Figure 6d–f, while the Ni layer retained a sound surface. Unlike the AMB substrates, see Figure 3d–f, the cracking in Ni–P on DBA was evident upon visual inspection. Figure 7 shows the SEM images of the surface of the Ni and Ni–P layers on a DBA substrate with Si_3_N_4_ at the initial state and after 1000 cycles. The surface of the Ni and Ni–P layers was roughened compared to the initial state. The Ni–P layer was cracked and the crack was thicker than that of the Ni–P layer on AMB substrate, see Figure 4d. Moreover, the crack completely separated the Ni–P layer and even the aluminum under the Ni–P layer spewed out through the crack of the Ni–P layer. The electroplated Ni layer with the higher fracture toughness showed a more stable thermal shock reliability.

**Figure 8:**
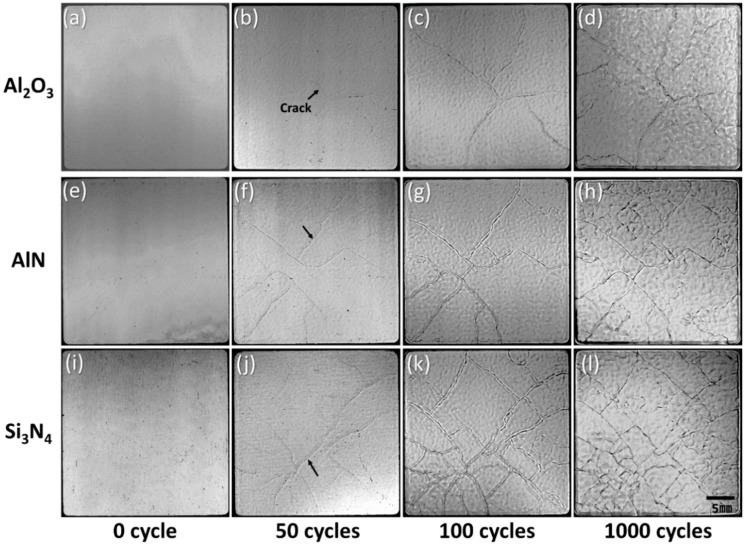
SAT observation of the surface morphology of the Ni–P layer on DBA substrates with Al_2_O_3_ (**a**–**d**), AlN (**e**–**h**), and Si_3_N_4_ (**i**–**l**) after different thermal shock tests: 0, 50, 100, and 1000 cycles.

**Figure 9:**
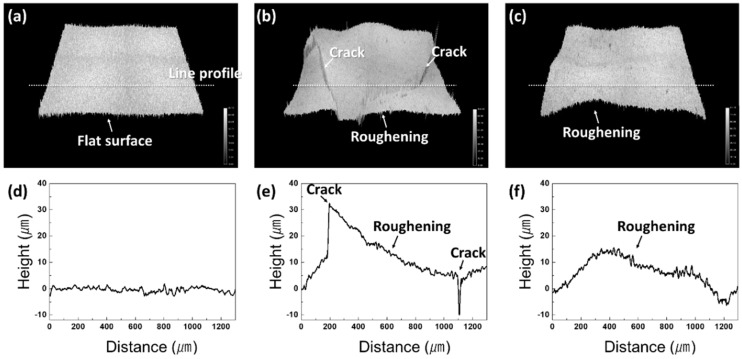
Laser surface observation of the Ni–P layer on DBA substrates with Si_3_N_4_ at the initial state (**a**) and after 1000 cycles (**b**,**c**). Local area with cracked (**b**) and roughened (**c**) area, and its roughness profile (**d**–**f**) measured by a line profile corresponding to (**a**–**c**), respectively.

To investigate the Ni–P crack evolution on DBA substrates with the three kinds of ceramic, SAT images were acquired. Figure 8 shows the evolution of the SAT surface images of the Ni–P layer on DBA substrates with Al_2_O_3_, AlN, and Si_3_N_4_ up to 1000 cycles. The Ni–P layer on all DBA substrates was cracked and roughened after 50 cycles. The cracks propagated and new cracks occurred up to 1000 cycles. The roughening also became more remarkable after 1000 cycles. Compared to the DBA substrates with AlN and Si_3_N_4_, fewer cracks were generated in the Ni–P layer on DBA substrates with Al_2_O_3_. The surface defects of the cycled specimens were analyzed by laser profile microscopy. Figure 9 shows the Ni–P layer on DBA substrates with Si_3_N_4_ at the initial state and after 1000 cycles at the location of the cracked and roughened surface. The surface of the Ni–P layer at the initial state was flat. After 1000 cycles, cracks occurred on the roughened surface of the Ni–P layer, see Figure 9b,e. The depth of the cracks was about 15–20 μm. This means that the crack separated not only the Ni–P layer, with a thickness of 7 μm, but also extended into the Al surface. The surface roughening of the Ni–P layer, see Figure 9c,f, was observed on the entire layer, regardless of cracks.

**Figure 10:**
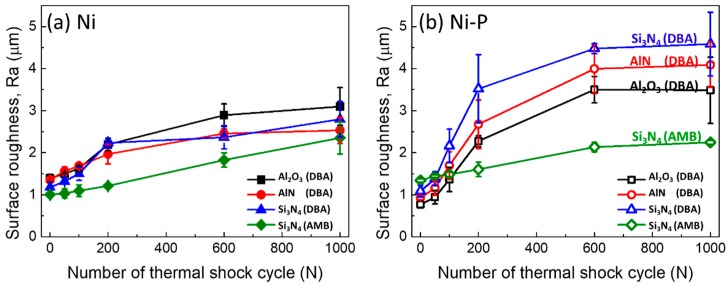
Variation in surface roughness of Ni (**a**) and Ni–P (**b**) layers on DBA/AMB substrates with Al_2_O_3_, AlN, and Si_3_N_4_ up to 1000 cycles.

Using laser profile microscopy, the roughness of the Ni and Ni–P layers on the DBA/AMB substrates was evaluated during the thermal shock cycling test, and the results are shown in Figure 10a,b, respectively. The AMB substrates with Al_2_O_3_ and AlN, which fractured after 10 cycles, were excluded from the roughness evaluation. The roughness of the Ni and Ni–P layers on the AMB substrates gradually increased. Regarding the DBA substrates, the roughness of all Ni layers increased by just a few micrometers, regardless of the type of ceramic plates. Ni–P layer roughness increased up to 600 cycles at different rates, and then remained nearly constant. Roughening in the Ni–P layer on a DBA substrate with Si_3_N_4_ ceramic was most remarkable, revealing that the Ni–P layer on DBA substrates with Si_3_N_4_ was most roughened among DBA substrates. Compared to the other ceramics in DBA, Si_3_N_4_ had the biggest CTE mismatch with Al metal, which induced the largest stress. Based on these results, the roughening in the Ni–P layer was probably related to thermal stress.

### 3.2. Cracking Mechanism of the Ni–P Layer

**Figure 11:**
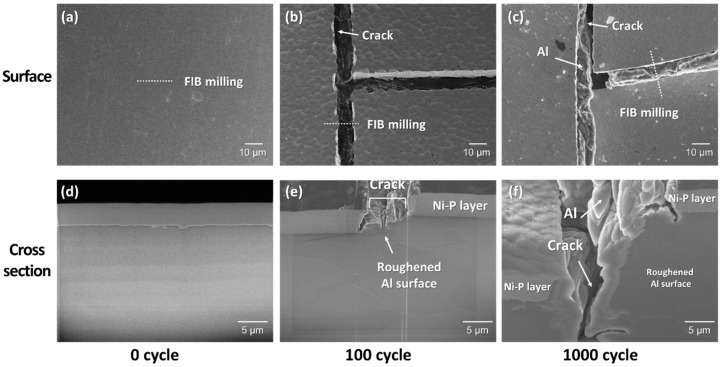
(**a**–**c**) SEM observation of the surface of the Ni–P layer on DBA substrates with Si_3_N_4_ at different thermal shock cycles of 0, 100, and 1000 cycles. (**d**–**f**) Focused ion beam (FIB)-milled cross-section image corresponding to (**a**–**c**), respectively.

Figure 11a,d show surface and cross-section images, respectively, of the Ni–P layer on DBA substrates with Si_3_N_4_ at an initial state. A FIB image of a cross-section was taken at an ion beam angle of 30°. The Ni–P layer was uniformly plated according to the flat Al surface morphology of the DBA substrates. Figure 11e,f shows the cross section of cracks in the Ni–P layer after 100 and 1000 cycles, respectively. The flat Al was deformed after 100 cycles. The crack in the Ni–P layer occurred just on the deformed Al surface. The deformation of Al metal in DBA substrates likely caused the cracking and roughening of the Ni–P layer. After 1000 cycles, the deformation of Al metal became serious. It caused deep cracking in the Ni–P layer and even spewed Al metal.

**Figure 12:**
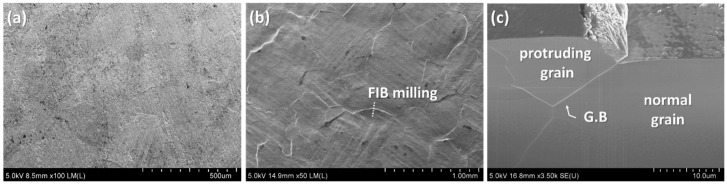
SEM observation of bare aluminum surface in DBA substrates with Si_3_N_4_ at the initial state (**a**) and after thermal shock tests of 100 cycles (**b**), and FIB-milled cross section (**c**) at the location of surface deformation.

To confirm the reasons for crack occurrences in the Ni–P layer on the DBA substrates, a bare DBA substrate with no plating layer was observed after a thermal shock cycling test. The flat aluminum surface at an initial state, as shown in Figure 12a, was significantly roughened after 100 cycles, see Figure 12b. Al roughening occurred according to the shape of an aluminum grain. Some grains protruded from an aluminum surface after cycle tests, as shown in Figure 12c. A grain boundary was observed under the roughened surface of aluminum, especially at the interface between the protruding grain and the nondeformed grain. Normally, grain boundary sliding (GBS) deformation of polycrystalline metal, one of the creep plastic deformation mechanisms, occurs under high temperatures and tensile/compressive stress [35]. During the thermal shock cycling test, Al metal on DBA was exposed to the most elevated temperature (250 °C) and also suffered from compressive and tensile stress that was caused by the CTE mismatch between aluminum and ceramic.

The deformation occurring in the Al layer, see Figure 12c, was considered as the GBS deformation because it met the conditions for GBS deformation occurrence at a high temperature. For AMB substrates, it was also reported that copper in AMB substrates is roughened through grain boundary sliding after thermal cycling from −55 to 250 °C [26], which is similar to the condition of the thermal shock test in this study. Metallic deformation can result from GBS deformation as well as simple homogeneous deformation. The observed Al GBS deformation and the referred Cu GBS deformation indicated that GBS might be one of the main deformation mechanisms of the metallic layer, resulting in the cracking of the Ni–P layer on the DBA/AMB substrates.

### 3.3. FEM Simulation

**Figure 13:**
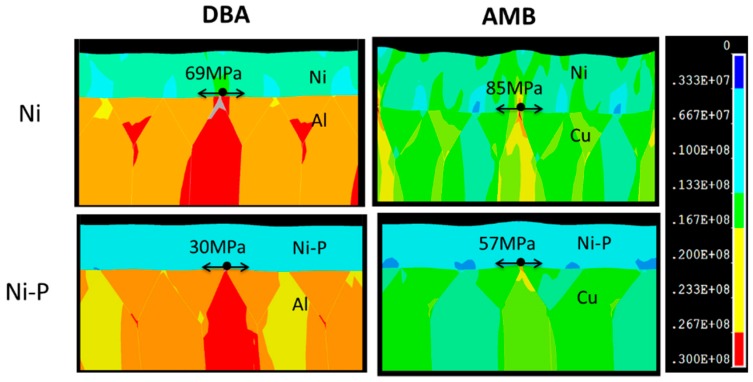
The maximum principal stress distribution for the Ni and Ni–P layers on DBA/AMB substrates with Si_3_N_4_ at 250 °C during the thermal shock cycling test.

Figure 13 shows the maximum principal stress distribution of DBA and AMB with the same Si_3_N_4_ plated with Ni and Ni–P layers. The FEM simulation of the maximum principal stress distribution was conducted at the thermal shock cycling test from −50 to 250 °C. In the case of DBA, the stress concentration at the interface between the polycrystalline Al grain layer and the plated Ni and Ni–P layers is shown in Figure 13a,c. The maximum stress was 69 and 30 MPa for the Ni- and Ni–P-plated layers, respectively. In the case of AMB, the maximum principal stress was also concentrated at the interface between the crystalline Cu layer and the Ni and Ni–P layers, as shown in Figure 13b,d, respectively. The maximum stress at the interface of Ni/Cu was also larger than that of Ni–P/Cu. This result may have been induced by the differences of materials properties between the Ni and Ni–P layers, especially Young’s modulus, as shown in Table 2. In addition, although the maximum stress at the interface between the Ni layer and both metal layers was larger than that which was between the Ni–P layer and both metal layers, the surface deformation and crack occurred at the Ni–P layer on DBA and AMB substrates, as shown in Figure 4d and Figure 7d. The cracking of the Ni–P layer might be explained by the differing fracture toughness between the Ni–P electroless and electroplated Ni layers. The fracture toughness of a crystalline Ni–P electroless plating layer is between 1.1 and 2.1 MPa·m^1/2^ [32,33,34], while an electroplated Ni layer is around 53 MPa·m^1/2^ [31]. High fracture toughness is related to crack initiation resistance and slow crack growth under the same stress conditions [36]. The ductile Ni layer with a higher fracture toughness likely suppressed crack initiation as well as growth by absorbing cracking energy. This is the reason why the electroplated Ni layer showed more crack resistivity during the thermal shock test. The FEM simulation result indicated that the fracture toughness of the plating layer on the DBA substrate was a key factor, which may influence crack generation and propagation.

Compared with the maximum stress that occurred at the DBA, the maximum stress increased in the AMB structure for both cases of plated layers; although, the aluminum in DBA had a bigger CTE mismatch with ceramic compared with copper in AMB. The stress changed from 69 to 85 MPa in the case of Ni plating and changed from 30 to 57 MPa in the case of the Ni–P plating substrate. The reason could be that since Al has a lower Young’s modulus as well as yielding stress, this led to stress release during the thermal shock test. The results also supported the experimental results, which demonstrated that the AMB substrate with Al_2_O_3_ and AlN ceramic fractured easily, as shown in Figure 3. In addition, it was also found that the stress distribution accumulated at the grain boundary and triple points in the Al and Cu layer. The reason for this may be a concentration of thermal stress at the grain boundaries and triple points due to material and geometrical singularities. As explained in Section 3.2, GBS deformation occurred in the Al and Cu layer along with the grain boundaries, thus leading to deformation and crack generation of the Ni–P layer plated on the DBA and AMB substrates.

## 4. Conclusions

In this work, a thermal shock cycling test of DBA/AMB substrates with three types of ceramic plates and two types of Ni metallization was presented. The material combination for high thermal shock resistance was confirmed. The failure mechanism for the Ni and Ni–P plating layers on the substrates was also discussed.

(1) AMB substrates with AlN and Al_2_O_3_ fractured after a thermal shock test of 10 cycles between −50 and 250 °C, while AMB with Si_3_N_4_ survived after a thermal shock test of 1000 cycles. On the other hand, all DBA substrates were not fractured until 1000 cycles. However, after 1000 cycles, the Ni–P electroless plating layer on the surviving DBA/AMB substrates became rough and cracked, regardless of the ceramic and metal type of substrates after 1000 cycles, while the Ni electroplating was not cracked. This is because the ductile Ni layer had a higher fracture toughness than the Ni–P layer, which could suppress crack generation by absorbing cracking energy.
(2) Beneath the cracked Ni–P layer on the DBA substrates, GBS deformation of Al was observed. It is considered that such deformation resulted in the cracking and roughening of the Ni–P layer. The thermal stress distribution simulation indicated that the tensile stress was concentrated at the interface between the polycrystalline grain layer and the plated Ni–P layer, along with the grain boundary. The results supported the cracking mechanism of the Ni–P layer by the GBS deformation of the metal.
(3) The Ni-plated AMB substrates with Si_3_N_4_ as well as the Ni-plated DBA substrates with Al_2_O_3_, AlN, and Si_3_N_4_ are promising material designs for high-temperature power substrates based on the proper ductility of the electroplated Ni layer.

## Figures and Tables

**Table 1:** Materials and dimensions of multilayered specimens.

Substrates			Metallization (Thickness: 7 μm)
Type	Metal (30 × 30 × 0.31 mm)	Ceramic (32 × 32 × 0.65 mm)	
DBA	Al	Al_2_O_3_	Ni electroplating or Ni–P electroless plating
		AlN	Ni electroplating or Ni–P electroless plating
		Si_3_N_4_	Ni electroplating or Ni–P electroless plating
AMB	Cu	Al_2_O_3_	Ni electroplating or Ni–P electroless plating
		AlN	Ni electroplating or Ni–P electroless plating
		Si_3_N_4_	Ni electroplating or Ni–P electroless plating

**Table 2:** Mechanical and thermal properties of the various materials in Ni- and Ni–P-plated DBA/AMB substrates.

Materials	Young’s Modulus (GPa)	Poisson’s Ratio	Coefficient of Thermal Expansion (µm·m^−1^·K^−1^)	Yield Strength (GPa)	Tensile Strength (MPa)	Fracture Toughness (MPa·m^1/2^)
Cu (001) [27,28]	75.7	0.28	17.7	0.7	-	
Cu (110) [27,28]	101.8	0.3	17.7	0.93	-	
Cu (111) [27,28]	123.4	0.3	17.7	1.2	-	
Al (001) [29]	63.7	0.34	21.3	0.59	-	
Al (110) [29]	72.59	0.34	21.3	0.52	-	
Al (111) [29]	76.1	0.34	21.3	0.51	-	
Al_2_O_3_	280	0.23	7.9	-	-	4
AlN	320	0.24	4.6	-	-	3
Si_3_N_4_	290	0.27	2.9	-	-	7
Ni [30,31]	220	0.31	14.1	0.08	500–1000	53
Ni–P [30,32,33,34]	50	0.31	13.0	0.23	50–150	1.1–2.1
